# Enhancing Geometric Fidelity of 3-Dimensional Electroanatomic Mapping During Open Chest Epicardial Radiofrequency Catheter Ablation

**DOI:** 10.1177/2324709619843948

**Published:** 2019-05-01

**Authors:** Gabriel E. Soto, Joseph G. Gibbons

**Affiliations:** 1 SoutheastHEALTH, Cape Girardeau, MO, USA; 2 Abbott Laboratories, Abbott Park, IL, USA

**Keywords:** electrophysiology, electroanatomic mapping, ablation, ventricular tachycardia, epicardial, open chest

## Abstract

Although electroanatomic mapping techniques have been previously applied to open chest epicardial ablation procedures, such efforts have often been limited by significant geometric distortions introduced by the need to use nonstandard mapping patch placements and by intrathoracic conductance changes introduced by having the pericardial space exposed. In this article, we present a case of a patient with recurrent hemodynamically unstable ventricular tachycardia who underwent a successful open chest epicardial ablation procedure with electroanatomic mapping in which geometric distortions were minimized by judicious placement of mapping patches and the use of a saline bath within the pericardial space.

## Introduction

Electroanatomic mapping (EAM) has revolutionized the field of modern interventional electrophysiology. Modern EAM systems allow clinicians to visualize complex anatomical relationships and catheter positions, and to track delivered ablation lesions while limiting a patient’s exposure to fluoroscopy and harmful radiation.^1-4^ EAM systems are usually utilized during endovascular ablation procedures, though they have also been successfully employed during epicardial ablation procedures performed utilizing minimally invasive transthoracic pericardial access techniques.^5-9^ In recent years, hybrid procedures have been described that combine epicardial access via a limited anterior thoracotomy or median sternotomy with EAM.^10,11^ However, such hybrid procedures—particularly those involving a median sternotomy—often result in significant geometric distortions in the generated electroanatomic maps due to the need to relocate mapping patches away from their standard anterior-posterior locations and the presence of air (effectively a nonconducting insulator) around the exposed heart that impairs the conduction of low-level currents utilized by EAM systems that depend on such currents for catheter localization.

We herein present a case report of a patient with recurrent ventricular tachycardia (VT) following coronary artery bypass grafting and a mitral valve replacement (MVR). The patient underwent an open chest epicardial catheter ablation in which an EAM system was successfully used using a novel irrigated saline bath configuration to facilitate mapping and lesion tracking in a situation where direct visualization of the myocardial VT substrate was not possible due to its posterobasal location and the patient’s hemodynamic instability during attempts at retracting the heart.

## Case Report

A 72-year-old male with new-onset heart failure and severe mitral regurgitation underwent coronary angiography, which demonstrated a 60% proximal-left anterior descending (LAD) stenosis, an 80% ostial-second diagonal (D_2_)-branch stenosis, a 90% proximal-first obtuse marginal (OM_1_) stenosis, an 80% mid-right coronary artery (RCA) stenosis, and a 95% distal-RCA stenosis; his left ventricular ejection fraction was 35% with posterobasal akinesis seen on ventriculography. A radionuclide uptake study showed viability of the inferolateral wall and anteroapical septum; the base of the posterior wall was not viable. A transesophageal echocardiogram confirmed severe eccentric mitral regurgitation. He electively underwent 4-vessel coronary artery bypass grafting consisting of a left internal mammary artery (LIMA) → LAD, saphenous vein graft (SVG) → D_2_, SVG → OM_1_, and SVG → RCA; he also underwent a MVR with a #29 bovine pericardial valve.

Postoperatively on the day of his surgery, he went into sustained VT, which on cardioversion degenerated into ventricular fibrillation and required cardiopulmonary resuscitation. He was ultimately successfully resuscitated, and was started on intravenous amiodarone, lidocaine, and β-blockers; he also had an intra-aortic balloon pump (IABP) inserted at the bedside. Over the next several days, he continued having multiple episodes of sustained and hemodynamically unstable VT (up to 5 per 24 hours) despite ongoing antiarrhythmic drug therapy (total amiodarone dose: >12 g); he also had a flail chest from multiple rib and sternal fractures sustained during his many episodes of cardiopulmonary resuscitation.

On the 12th postoperative day, a decision was made to proceed with a combined sternal repair and open chest epicardial catheter ablation in order to stabilize the patient’s condition.

## Methods

### Patient Preparation

**Figure 1.**
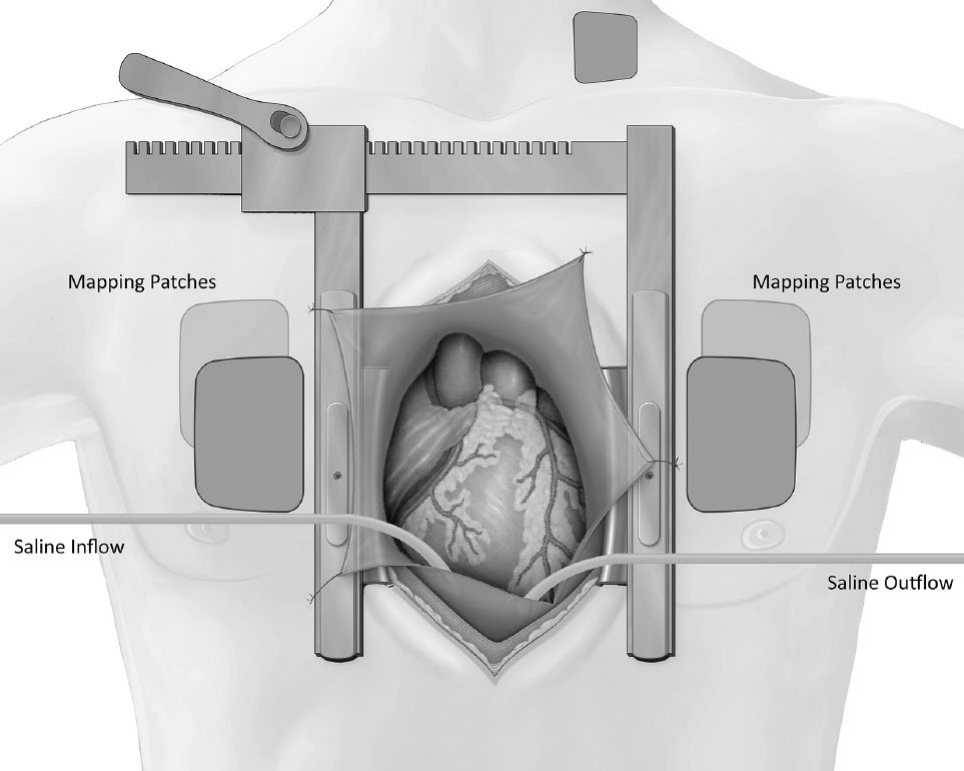
Schematic representation of NavX skin navigation patches relative to the median sternotomy incision. The anteroposterior and lateral patches were offset by approximately 45° along the patient’s craniocaudal axis from their usual positions. Inflow and outflow lines maintained a stable level of saline fluid within the pericardial space.

The patient was brought to the electrophysiology laboratory from the cardiothoracic intensive care unit (CT-ICU), intubated, and on mechanical ventilatory support. Electrocardiography limb leads were placed in the usual position, but leads V_1_-V_6_ were placed posteriorly to allow for surgical access to the anterior chest wall. In order to accommodate the median sternotomy, the 4 torso mapping patches were rotated 45° along the patient’s craniocaudal axis from their usual positions. This allowed for maintenance of the relative orthogonal relationship of each patch pair (ie, anterior/posterior and right-lateral/left-lateral patches) with respect to one another; the neck and thigh patches remained in their usual locations (Figure 1). The patient’s chest, abdomen, and groin regions were subsequently prepared and sterilely draped in the usual manner. Femoral venous access was obtained using the modified Seldinger technique for placement of diagnostic catheters.

### Electroanatomic Mapping

A deflectable decapolar catheter (St Jude Medical, Inc, St. Paul, MN) and quadpolar catheter (C R Bard, Inc, Lowell, MA) were positioned within the coronary sinus and at the right ventricular apex, respectively. EAM was performed utilizing EnSite Velocity (St Jude Medical, Inc, St Paul, MN), which employs a low-level 8.128 kHz current through the orthogonally located skin patches. The recorded voltage and impedance at each catheter’s electrodes generated from this current allows their distance from each skin patch, and ultimately, their location in space, to be triangulated with the help of a reference electrode.

In order to provide a stable conducting medium around the exposed myocardium, the pericardial space was continuously irrigated with normal saline prewarmed to 37°C and delivered via sterile vinyl tubing. A low level of continuous suction was applied via an outflow line to maintain a stable fluid level within the open pericardial cavity. Validation was performed only after the chest had been opened to its fullest excursion and the pericardial space had been filled with saline.

A Safire TX 8 mm tip catheter (St Jude Medical, Inc, St Paul, MN) was placed within the open pericardial space, below the fluid level; a 3D-electroanatomic shell of the left and right ventricles were created using EnSite Velocity only using geometry collected from the distal bi-pole of the catheter. The geometries of the right and left ventricles were divided based on the observed courses of the LAD and right posterior descending coronary arteries as determined by direct visual inspection. Voltage mapping was performed using a scare range of 1.5 mV to 0.3 mV.

### Cardiac Ablation

All ablations were carried out utilizing power settings of 70 to 90 Watts and a temperature cutoff of 65°C to 80°C. The efficacy of delivered lesions was monitored by the following: (1) loss of local electrograms and (2) loss of pacing capture. Lesion locations were tracked utilizing EnSite Velocity.

## Results

### Initial Operative Findings

After reopening of the sternum, inspection demonstrated obvious dehiscence and fractures between the manubrium, sternal body, and xiphoid process, as well as the presence of a large hematoma. All wires were removed and the clot evacuated.

Manual inspection of the heart demonstrated a large well-circumscribed area of infarct along the posterobasal wall; however, attempts to maintain exposure of the posterior wall via manual retraction of the heart apex were precluded by severe hypotension necessitating intravenous vasopressor support, presumably due to significantly impaired left ventricular filling dynamics with apical traction in the setting of the patient’s recent MVR.

### Electrophysiology Study and Electroanatomic Mapping

**Figure 2.**
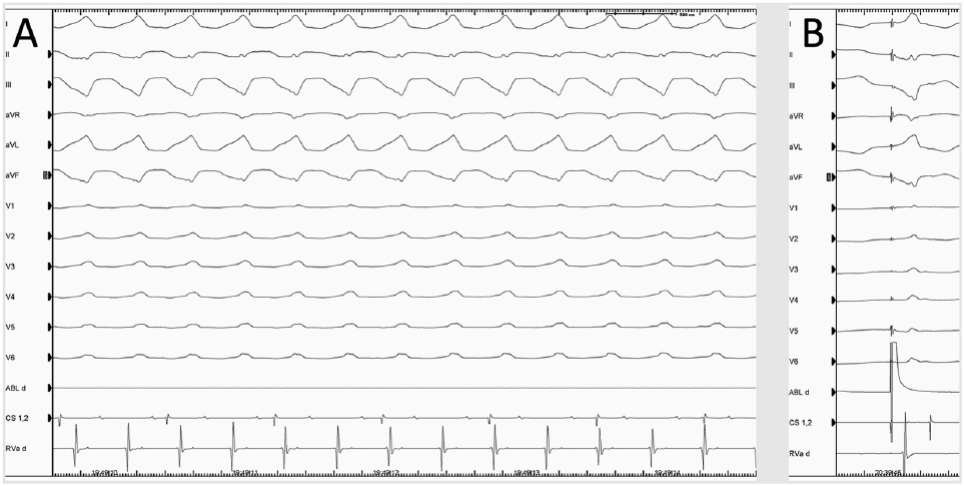
(A) Surface ECG of ventricular tachycardia at a cycle length of 380 ms. (B) Pace mapping: QRS morphology during pacing at 600 ms at the targeted ablation site.

Sustained VT at a cycle length of 380 ms was readily induced via overdrive ventricular pacing at 280 ms from the right ventricular apex (Figure 2A). The cycle length of the induced VT matched that of the patient’s documented clinical VT. The patient tolerated the VT extremely poorly from a hemodynamic standpoint, precluding attempts at entrainment mapping. DC cardioversion at 10 J delivered via intracardiac paddles was used to restore sinus rhythm; however, the patient exhibited several minutes of pulseless electrical activity during which an epinephrine infusion was initiated and direct cardiac massage was performed until return of spontaneous circulation.

**Figure 3.**
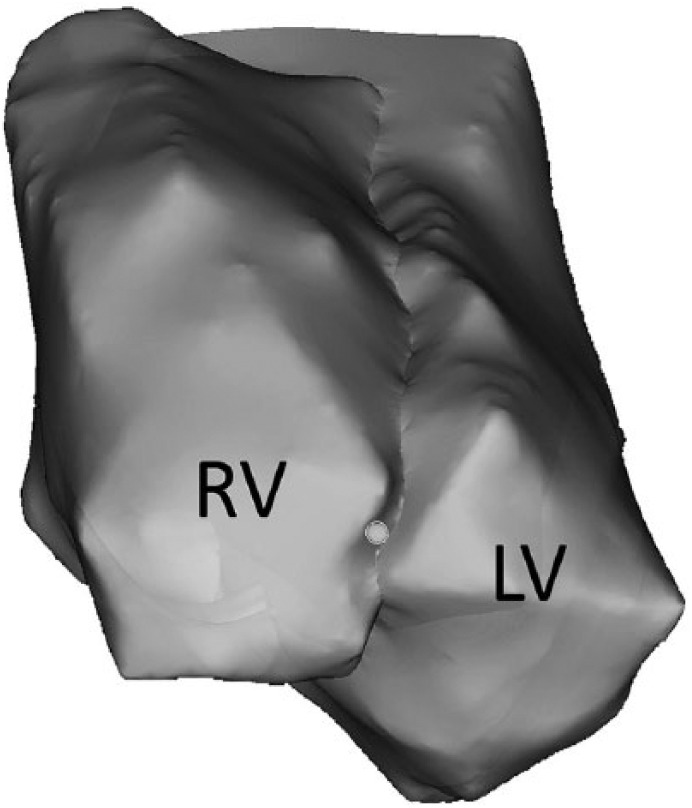
Three-dimensional surface geometries of the right ventricle (RV) and left ventricle (LV).

Given the patient’s poor hemodynamic tolerance of his VT, a decision was made to pursue pace mapping along the edge of the identified posterior wall scar for what was presumed to be re-entrant VT. As the patient did not tolerate retraction of the heart apex for visualization of the posterior wall, EAM was performed with the heart exposed but in situ utilizing EnSite Velocity. Three-dimensional geometries of the left and right ventricles were created (Figure 3) as described in the Methods section.

**Figure 4.**
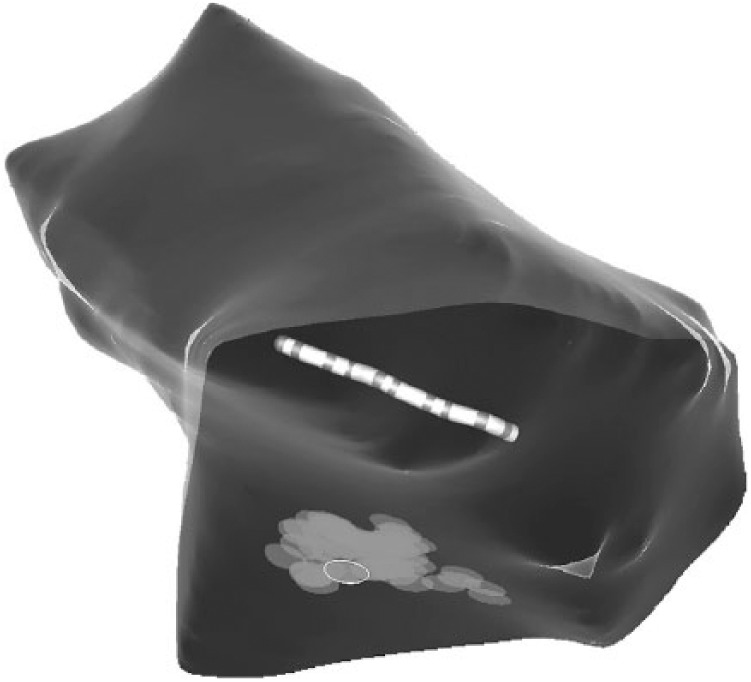
Ablation lesions (circles) delivered along the posterobasal wall. The position of the deflectable decapolar catheter within the coronary sinus can be seen.

Pace mapping along the edge of the posterobasal infarct zone identified a putative breakout site for the patient’s ventricular tachycardia: the QRS morphology during pacing was a match in all 12 leads (Figure 2B). A series of radiofrequency ablation lesions were delivered at this site and in the immediate vicinity extending into the scar region (Figure 4). *Total radiofrequency application time*: approximately 15 minutes.

On subsequent induction attempts, another faster VT with a distinct QRS morphology was induced, which rapidly degenerated into ventricular fibrillation. Cardioversion attempts at 10 J and 20 J (delivered via intracardiac paddles) were unsuccessful; a third cardioversion attempt at 30 J was successful in restoring sinus rhythm. Once again, several minutes of direct cardiac massage was performed until return of spontaneous circulation. No further attempts at induction were performed.

### Case Conclusion, Clinical Course, and Follow-up

The patient underwent placement of an epicardial lead prior to sternotomy repair and chest closure. He was transferred back to the CT-ICU with an IABP in place. He had a protracted recovery complicated by renal failure necessitating dialysis and by ventilator-associated pneumonia necessitating prolonged ventilator support; however, he had no further episodes of VT during his hospital stay, and after being weaned from ventilator support he ultimately underwent placement of a Unify 3231-40Q CRT-D biventricular pacemaker/implantable cardioverter-defibrillator (St Jude Medical, Inc, St Paul, MN) on postoperative day 68. He was ultimately discharged on postoperative day 84. At 1-, 3-, 6-, and 12-month follow-up, the patient had experienced no recurrent episodes of ventricular tachycardia or ventricular fibrillation. Follow-up echocardiography demonstrated a modest improvement in his left ventricular ejection fraction to 40%.

## Discussion

The majority of cardiac ablations for treatment of ventricular tachycardia are performed via endovascular approaches, although a subset of patients will require epicardial ablation for successful treatment. When the latter is required, subxiphoid access to the pericardial space via percutaneous techniques or minimally invasive surgical access has been demonstrated to be safe and effective, and generally does not pose any special challenges from the standpoint of being able to employ EAM to facilitate mapping and ablation.^5-9^

For a subset of patients such as the one presented herein, a hybrid open chest procedure via a median sternotomy may be indicated. Patel et al published the largest such series of patients to date, in which 5 patients with end-stage heart failure underwent an open chest electrophysiology study with epicardial mapping during the period of LVAD placement.^11^ Although EAM was attempted, they noted that their efforts resulted in “distorted maps that were not reliable,” and the operators relied heavily on prior endocardial maps that had been created in 4 out of the 5 patients. The decision to place the anterior and posterior mapping patches near the axilla to allow for complete exposure of the thoracic cavity may have contributed to these distortions.^11^

In this study, the creation of geometries with a high degree of anatomic fidelity and minimal geometric distortion was created by the following: (1) applying all 4 torso mapping patches such that they were rotated 45° along the patient’s craniocaudal axis from their usual positions, allowing for maintenance of the relative orthogonal relationship of the patches to each other; (2) only using geometry collected from the distal bi-pole of the mapping catheter; and (3) the creation of a stable conductance medium by bathing the exposed pericardial space in saline. A secondary benefit of the saline bath was the absence of difficulties associated with high impedances during ablation as reported in other studies.^10^ Although endocardial mapping was not performed during the course of this study, the otherwise standard system setup employed should have allowed for concurrent endocardial geometries and maps to be generated if needed.

The techniques presented herein will hopefully add to the armament of tools that electrophysiologists have at their disposal when tackling otherwise difficult VT ablation cases.
